# From Transcriptome to Therapy: The ncRNA Revolution in Neurodevelopmental Disorders

**DOI:** 10.3390/brainsci16010017

**Published:** 2025-12-23

**Authors:** Jiayi Zhao, Shanshan Li, Xin Jin

**Affiliations:** 1 Department of Life Sciences, Faculty of Natural Sciences, Imperial College London, London SW7 2AZ, UK; 2 School of Medicine, Nankai University, Tianjin 300071, China; 3 Academy for Advanced Interdisciplinary Studies, Nankai University, Tianjin 300071, China

**Keywords:** non-coding RNA, neurodevelopmental disorders, transcriptomics, biomarkers, RNA therapeutics

## Abstract

Neurodevelopmental disorders (NDDs) such as autism spectrum disorder (ASD), attention-deficit/hyperactivity disorder (ADHD), and intellectual disability (ID) arise from disruptions of molecular programmes that coordinate neurogenesis, synaptogenesis, and circuit maturation. While genomic studies have identified numerous susceptibility loci, genetic variation alone accounts for only part of disease heritability, underscoring the importance of post-transcriptional and epigenetic regulation. Among these regulatory layers, non-coding RNAs (ncRNAs), including microRNAs (miRNAs), long non-coding RNAs (lncRNAs), circular RNAs (circRNAs), PIWI-interacting RNAs (piRNAs), and transfer RNA-derived small RNAs (tsRNAs), have emerged as central modulators of neural differentiation, synaptic plasticity, and intercellular signalling. Recent multi-omics and single-cell studies reveal that ncRNAs fine-tune chromatin accessibility, transcriptional output, and translation through tightly integrated regulatory networks. miRNAs shape neurogenic transitions and circuit refinement; lncRNAs and circRNAs couple chromatin architecture to activity-dependent transcription; and tsRNAs and piRNAs extend this regulation by linking translational control to epigenetic memory and environmental responsiveness. Spatial transcriptomics further maps ncRNA expression to vulnerable neuronal and glial subtypes across cortical and subcortical regions. Clinically, circulating ncRNAs, especially those packaged in extracellular vesicles, exhibit stable, disease-associated signatures, supporting their potential as minimally invasive biomarkers for early diagnosis and patient stratification. Parallel advances in RNA interference, antisense oligonucleotides, CRISPR-based editing, and vesicle-mediated delivery highlight emerging therapeutic opportunities. These developments position ncRNAs as both mechanistic determinants and translational targets in NDDs, offering a unifying framework that links genome regulation, environmental cues, and neural plasticity, and paving the way for next-generation RNA-guided diagnostics and therapeutics.

## 1. Introduction

NDDs, including autism spectrum disorder (ASD), attention-deficit/hyperactivity disorder (ADHD), and intellectual disability (ID), represent a major global health challenge due to their early onset, lifelong persistence, and profound impact on cognitive, social, and adaptive functioning. Their high prevalence and complex aetiology place a considerable emotional and socioeconomic burden on affected individuals, families, and healthcare systems. Although extensive genetic studies have identified numerous risk loci, these findings alone fail to explain the full spectrum of phenotypic variability observed in patients, underscoring the need to explore additional layers of molecular regulation beyond DNA sequence variation [1]. This gap underscores the need to investigate regulatory layers beyond DNA sequence variation, particularly those that govern transcriptional, post-transcriptional, and translational dynamics during brain development.

Recent work highlights non-coding RNAs (ncRNAs), including microRNAs (miRNAs), long non-coding RNAs (lncRNAs), circular RNAs (circRNAs), PIWI-interacting RNAs (piRNAs), and tRNA-derived small RNAs (tsRNAs), as critical regulators of these developmental programmes. These ncRNAs orchestrate neurodevelopment through conserved biochemical pathways: (1) chromatin and epigenetic modification, (2) transcriptional regulation, (3) post-transcriptional control of mRNA stability and translation, (4) modulation of local protein synthesis in axons and synapses, and (5) coupling neural activity to structural plasticity. Dysregulation of these pathways contributes to the disruption of neuronal connectivity, excitatory–inhibitory balance, and synaptic refinement that characterise many NDDs.

While diverse ncRNA alterations have been described across neurological conditions, the present review focuses specifically on neurodevelopmental mechanisms. This clarification addresses conceptual ambiguity noted in the previous literature and ensures a coherent neurodevelopmental focus. Multi-omics profiling, including bulk RNA-seq, small RNA-seq, single-cell and spatial transcriptomics, has revealed that ncRNAs display remarkable cell-type- and region-specific expression in the developing cortex, hippocampus, striatum, and cerebellum. Importantly, cohort studies, clinical studies, and exploratory studies report varying degrees of reproducibility.

Because ncRNAs integrate genetic, metabolic, and environmental signals, including maternal stress, hypoxia, air pollution, immune activation, and activity-dependent synaptic cues, they form a mechanistically informative layer linking risk exposure to aberrant neurodevelopmental outcomes. ncRNA-dependent regulation provides a compelling framework for understanding how multifactorial risks converge on shared biochemical pathways that disrupt neurogenesis and circuit maturation. Accordingly, this review synthesises current evidence on how each major ncRNA class shapes neurogenesis, neuronal migration, synaptic formation, dendritic/axonal development, and circuit refinement, and how their dysregulation contributes to ASD, ADHD, and ID. We prioritise mechanistic pathways, reproducible findings, and experimental systems with demonstrable functional consequences, including gain/loss-of-function models, organoids, and single-cell regulatory networks. We also highlight limitations, such as inconsistent replication across cohorts, variability in extracellular vesicle (EV)-derived ncRNA studies, and the need for functional validation of computationally predicted ncRNA–mRNA interactions.

**Figure 1:**
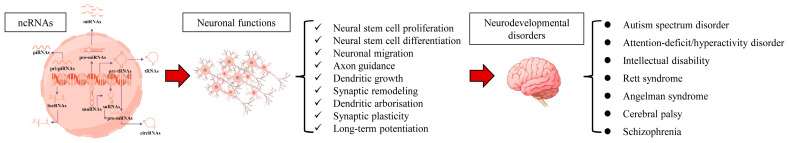
Overview of how ncRNA dysregulation disrupts neuronal functions and contributes to neurodevelopmental disorders. This schematic illustrates the multilayered roles of ncRNAs, including miRNAs, lncRNAs, circRNAs, piRNAs, tsRNAs, and snoRNAs, in neural development. **Left** panel: ncRNA species within neurons regulate transcription, chromatin state, RNA stability, and translation through interactions with RNA-binding proteins, ribonucleoprotein complexes, and ceRNA networks. **Middle** panel: Through these mechanisms, ncRNAs exert essential control over multiple neuronal functions, including neural stem-cell proliferation and differentiation, neuronal migration, axon guidance, dendritic growth, synaptic remodelling and arborisation, synaptic plasticity, and long-term potentiation. **Right** panel: Disruption of these ncRNA-dependent regulatory programs contributes to the pathophysiology of diverse neurodevelopmental disorders, including autism spectrum disorder, attention-deficit/hyperactivity disorder, intellectual disability, Rett syndrome, Angelman syndrome, cerebral palsy, and schizophrenia.

Together, these refinements define the scope of this review: to provide a neurodevelopment-focused evaluation of ncRNAs, to clarify how RNA-based regulatory networks shape normal brain development and contribute to NDD pathogenesis (Figure 1).

## 2. MicroRNAs in Neurodevelopmental Disorders

### 2.1. Fundamental Roles of miRNAs in Neurodevelopment

The temporal and spatial choreography of miRNA expression mirrors the sequential milestones of brain development, from neural stem cell proliferation and differentiation to synaptic maturation and circuit refinement. During embryonic and early postnatal stages, canonical miRNAs such as miR-9, miR-124, and miR-132 orchestrate neurodevelopmental transitions by repressing multiple mRNA targets in a coordinated manner, thereby ensuring the precise timing of neuronal lineage commitment, axonal growth, and dendritic patterning [2]. As neural circuits mature, these miRNAs act as molecular integrators that translate transcriptional, epigenetic, and environmental cues into finely tuned post-transcriptional programmes governing synaptic plasticity and network stability.

Exosome-encapsulated miRNAs, including miR-21, miR-132, and miR-134, are released by neurons and glia as vesicle-borne messengers that traverse the blood–brain barrier and influence neuroimmune and metabolic signalling [3]. Their stability in extracellular fluids positions them as both local regulators of neural homeostasis and systemic biomarkers of neurodevelopmental health. Activity-dependent transcription factors such as CREB and MEF2 directly regulate DROSHA/DICER processing [2], coupling miRNA biogenesis to synaptic excitation and chromatin state. miRNAs modulate several core biochemical axes, including chromatin remodelling, mitochondrial metabolism, neurotrophic (BDNF–TrkB) signalling, and cytoskeletal MAPK–CRMP2 pathways, each of which is systematically summarised in Table 1.

### 2.2. miRNAs and Autism Spectrum Disorder (ASD)

ASD is characterised by extensive dysregulation of miRNA networks across cortical and subcortical regions, peripheral biofluids, and cellular models. Integrated mRNA–miRNA profiling of human post-mortem cortex reveals that mitochondrial and oxidative-phosphorylation genes are co-regulated by miR-181a-5p and miR-34a, defining a metabolic signature of neuronal stress [4].

Maternal hypoxia studies revealed that the miR-23b/27b/24 cluster is altered in the offspring brain, suggesting intergenerational epigenetic transmission [5]. Network-based analyses further show that miRNA targetomes overlap with high-confidence ASD genes such as CHD8, linking miRNA dysregulation to Notch-regulated progenitor differentiation [18].

Functional in vivo evidence demonstrates that loss of exosomal miR-215-5p activates the NEAT1/MAPK1/p-CRMP2 cascade, impairing synaptic structure and social behaviour in VPA-exposed mice [6]. Plasma miRNA studies identify miR-195-5p and miR-499a-5p as clinically relevant candidates associated with symptom severity and neuroanatomical variation [8,9]. Systems biology identifies miR-21 as a pleiotropic node linking vascular stress with neurodevelopmental vulnerability [9], whereas computational analyses implicate miR-146a, miR-181, and miR-21 in chromatin-level epigenetic regulation [10].

Depletion of AmnSINE1 repeat-derived transcripts in ASD cortex [19] implies that miRNA–retrotransposon interactions contribute to transcriptomic instability during development. In cellular and animal models, BTBR mice show consistent downregulation of prefrontal miRNAs involved in excitatory–inhibitory balance [20], and MEF2C-haploinsufficient hiPSC neurons exhibit network hyperexcitability reversible by NMDA receptor modulation [21]. Therapeutic restoration of miR-137 rescues metabolic and behavioural phenotypes [22]. Complementary plasma studies identify developmental shifts in miR-4433b-5p, miR-15a-5p, miR-335-5p, and miR-1180-3p, collectively yielding an AUC of 0.936 for early diagnosis [23]. Although several ASD-associated miRNAs have been replicated across independent cohorts, others, particularly those derived from rodent models, require translational validation.

### 2.3. miRNAs and Attention-Deficit/Hyperactivity Disorder (ADHD)

ADHD arises from disrupted molecular programmes that coordinate neurotransmission, neurotrophic signalling, and circadian rhythm. Multi-omics analyses reveal miRNA-regulated networks controlling synaptic transmission, dopamine metabolism, and clock-gene expression [24]. In six European birth cohorts, 29 blood miRNAs correlate with hyperactivity and three with inattention, capturing behavioural variability consistent with clinical phenotypes [25].

Case control studies identify downregulation of miR-34c-3p and miR-138-1, both targeting the BDNF pathway, accompanied by compensatory increases in serum BDNF [11]. Mechanistic studies delineate converging miRNA circuits governing synaptic plasticity and attentional control. The lncMALAT1–miR-141-3p/200a-3p–NRXN1 axis modulates synaptic-adhesion gene expression, impairing learning and memory in ADHD models [26]. In environmental paradigms, the miR-130/SNAP-25 pathway regulates presynaptic machinery in the anterior cingulate cortex and mediates lead-induced attentional deficits [12].

Extracellular-vesicle profiling in adolescents identifies miRNA clusters overlapping with depression and anxiety yet retaining ADHD-specific patterns [27]. Moreover, sex-biased miRNA expression in the developing mouse brain [28] offers a plausible explanation for the higher prevalence of ADHD in males and the heterogeneity of symptom expression. Clinically, circulating miRNAs such as miR-26b-5p, miR-185-5p, and miR-142-3p have emerged as reproducible predictors of ADHD diagnosis and treatment responsiveness [13,14,15]. Their peripheral stability and correlation with central neurotransmission pathways underscore their potential as non-invasive biomarkers for clinical stratification and longitudinal monitoring. These findings establish miRNAs as integrative regulators of dopaminergic, glutamatergic, and neurotrophic networks, bridging molecular neurobiology with translational psychiatry in ADHD.

### 2.4. Cross-Disorder Roles of miRNAs in Neurodevelopmental Impairment

The influence of miRNA dysregulation extends beyond ASD and ADHD. In Rett syndrome, modulation of miRNA–X-inactivation dynamics restores MECP2 dosage [29]. In perinatal hypoxia–ischaemia, bone-marrow-mesenchymal-stem-cell-derived exosomal miRNAs rescue cortical neurons and reduce gliosis, providing proof-of-concept for cell-free RNA therapies in cerebral palsy [30]. Glial miRNA signalling is exemplified by astrocyte-derived miR-483-5p and miR-138-5p, which regulate neuronal gene expression via extracellular vesicles [16]. miR-34a-5p-mediated suppression of SIRT1 links metabolic stress to ferroptotic vulnerability in hippocampal neurons [17].

While some of these pathways overlap with mechanisms implicated in neurodegeneration, they are included here only where they enhance understanding of developmental vulnerability. At a systems level, NR4A2 integrates miRNA regulatory inputs across neurodevelopmental and psychiatric dimensions [31]. Together, these findings highlight miRNAs as nodal regulators of chromatin, metabolism, circuitry, and intercellular communication.

Although extensive studies implicate miRNAs in synaptic development, metabolic regulation, and neuroimmune pathways, most available evidence remains correlative rather than causal. The convergence of miRNA–target networks on chromatin remodelling, mitochondrial function, and excitatory–inhibitory balance suggests that miRNAs function as integrators rather than isolated regulators. However, the field still lacks perturbation-based, cell-type-specific studies that can define hierarchical relationships among miRNAs, their targets, and circuit-level phenotypes. Future work using CRISPR-based miRNA editing, live-cell reporters, and single-cell perturb-seq will be critical for determining whether miRNA dysregulation is a primary driver or a downstream amplifier of neurodevelopmental pathology.

## 3. Long Non-Coding RNAs in Neurodevelopmental Disorders

### 3.1. Overview of lncRNA Biology in the Nervous System

lncRNAs comprise a heterogeneous class of transcripts (>200 nt) that regulate gene expression at epigenetic, transcriptional, and post-transcriptional levels. In the nervous system, lncRNAs direct neurogenesis, synaptogenesis, and circuit maturation through cis and trans mechanisms—recruiting chromatin modifiers, modulating transcriptional machinery, and scaffolding ribonucleoprotein complexes or ceRNA networks to fine-tune local translation and decay. Cross-disorder analyses highlight lncRNAs as central gatekeepers of neural homeostasis [32].

Abundant transcripts such as NEAT1, MALAT1, and H19 orchestrate activity-dependent transcription, synaptic organization, and neuroinflammatory signalling, with region- and stage-specific isoforms enabling spatiotemporal precision. Advances in single-cell and total-RNA sequencing reveal lineage-restricted expression programs in neural progenitors and differentiated neurons, positioning lncRNAs as molecular nodes that couple chromatin architecture to transcriptional and synaptic plasticity [33]. These observations situate lncRNAs as architectural elements of the neural transcriptome, integrating chromatin remodelling, transcriptional control, and RNA-processing pathways (Table 2).

### 3.2. lncRNAs Regulation in Autism Spectrum Disorder

Converging evidence identifies lncRNAs as key intermediates linking genetic susceptibility, environmental stress, and synaptic dysfunction in ASD. In a landmark study, NEAT1 exacerbated ASD-like behaviours in valproic-acid-exposed mice by recruiting YY1 to the UBE3A promoter, enhancing transcription and promoting neuroinflammation and oxidative stress [34]. This axis intersects with miRNA-mediated regulation: reduced exosomal miR-215-5p disinhibits the NEAT1/MAPK1/p-CRMP2 cascade, yielding parallel behavioural and synaptic phenotypes [6]. These connections position NEAT1 as a nodal regulator coupling environmental perturbation to transcriptional imbalance.

Beyond single transcripts, population-scale profiling demonstrates broad lncRNA perturbation. Peripheral blood analyses reveal altered PCAT-29, LINC-PINT, lincRNA-p21, lincRNA-ROR, and PCAT-1 [35]. Independent plasma studies identify circulating LINC00662, LINC00507, and LINC02259, implicating immune and synaptic pathways [36].

Mechanistic modelling emphasizes strong cell-type specificity. Using causal inference on human single-nucleus RNA-seq, glial- and excitatory neuron-restricted hubs, such as SOX2-OT and MIR155HG, show high ASD-gene connectivity [37]. Cross-species analyses, in Microtus ochrogaster, for example, highlight behavioural-state-dependent lncRNA engagement overlapping human psychiatric-risk modules [49].

Discrete lncRNA–protein and lncRNA–miRNA axes further refine the mechanism. The H19/miR-484 pathway tracks ASD severity and implicates oxidative and mitochondrial stress responses [38]. The CHD8–SINEUP RNA interaction exemplifies translational up-regulation: SINEUP restores protein synthesis and rescues CHD8-suppression phenotypes without altering mRNA abundance [39]. Genetic association connects dysregulated Csnk1a1p to kinase-mediated neurodevelopmental signalling [40]. Together, these findings define ASD as a disorder of transcriptional and post-transcriptional instability, in which lncRNAs modulate chromatin accessibility, synaptic gene expression, neuroinflammatory tone, and translational control (Table 2).

### 3.3. lncRNAs Regulation in Attention-Deficit/Hyperactivity Disorder

Mounting multi-omic, functional, and genetic data support lncRNA involvement in ADHD, particularly across circadian, synaptic, and cognitive domains. Peripheral profiling identifies rhythm-linked lncRNAs HULC and UCA1 as significantly elevated in ADHD independent of diurnal preference, suggesting disruption of lncRNA–clock feedback [41]. Mechanistically, the lncMALAT1–miR-141-3p/200a-3p–NRXN1 axis regulates synaptic adhesion and constrains learning and memory capacity [26], consistent with synaptic-plasticity deficits underlying cognitive symptoms.

A paracentric inversion disrupting SHANK2 and neighbouring LINC02714 was identified in an individual with ADHD [42]. Association analyses link RNF219-AS1 to ADHD behavioural phenotypes and white-matter microstructure [43]. An SNP within BC200, a translational regulator, associates with ADHD and other psychiatric disorders [44]. These findings span case studies, moderate-scale association analyses, and functional models; although mechanistically compelling, many require larger replication cohorts. Thus, lncRNAs appear to operate at the intersection of circadian regulation, synaptic adhesion, and structural-genomic integrity—translating regulatory imbalance into attentional and behavioural dysregulation.

### 3.4. lncRNA Dysfunction in Monogenic and Rare Neurodevelopmental Syndromes

LncRNA pathology extends to monogenic and rare syndromes where the non-coding element itself is causal. Deletion of CHASERR disrupts cis repression of CHD2, producing excessive CHD2 expression and a severe early-onset disorder with cortical atrophy and hypomyelination—establishing lncRNA haploinsufficiency as a direct pathogenic mechanism [45]. In Rett syndrome, lncRNA regulation surfaces as both a disease driver and therapeutic target. Modulation of microRNA-dependent X-chromosome inactivation alleviates Rett-like phenotypes [29], while NEAT1 coordinates proteostasis and mRNA localization in neurons, shaping autophagy and stress resilience [50]. Therapeutic platforms are beginning to leverage lncRNA biology directly. In Angelman syndrome, silencing of the paternal UBE3A allele by UBE3A-ATS can be reversed: an AAV-delivered dCas9 construct suppresses UBE3A-ATS transcription, unsilences paternal UBE3A and rescues behavioural deficits in mice—an allele-specific CRISPR interference proof-of-principle that avoids double-strand breaks [46]. Additional lncRNA-dependent mechanisms link to cognition and learning: dysregulation of the MNK–SYNGAP1 axis perturbs synaptic signalling [48], and lncRNA SPA, a splicing regulator during neural differentiation, is modulated by FUBP1, whose dual effects on SPA maturation underscore the intricacy of RNA-processing control [47]. These monogenic examples are included solely where they clarify fundamental principles of lncRNA dosage, RNA processing, and chromatin regulation relevant to neurodevelopmental vulnerability.

lncRNA biology in neurodevelopment remains conceptually rich but experimentally under-validated. While single-cell datasets increasingly highlight cell-type-restricted lncRNA modules, functional causality has been established for only a small subset, such as NEAT1, H19, and UBE3A-ATS. The diversity of lncRNA mechanisms, chromatin recruitment, transcriptional modulation, scaffolding, and ceRNA activity indicates that lncRNAs represent a multilayered regulatory axis rather than a single pathway. A major unresolved question concerns how lncRNA structure, localization, and interacting proteins determine their mechanistic specificity. Systematic perturbation strategies and structure–function mapping will be essential for translating lncRNA findings into therapeutic applications.

## 4. Circular RNAs in Neurodevelopmental Disorders

### 4.1. Biological Features of circRNAs in the Nervous System

circRNAs are covalently closed, single-stranded transcripts generated by back-splicing, in which a downstream 5′ splice donor joins an upstream 3′ acceptor. This circular topology renders circRNAs resistant to exonucleolytic degradation, endowing them with exceptional molecular stability and enabling their preferential accumulation in post-mitotic tissues such as the brain. CircRNAs are evolutionarily conserved and exhibit striking spatial and temporal specificity, with pronounced enrichment at synapses—features that implicate them in activity-dependent gene regulation, neuronal differentiation, and synaptic plasticity [32].

Functionally, circRNAs serve as multi-layered regulators of gene expression. They function as miRNA sponges, RBP scaffolds, transcriptional modulators, and in an increasing number of cases—templates for active translation in neurons. Several neuronal circRNAs, including circZNF609, circMbl, and circAβ-a, contain internal ribosome entry sites (IRES) or m^6^A-dependent translation initiation motifs that recruit ribosomes under physiological conditions. Ribosome profiling and mass spectrometry have confirmed the production of circRNA-derived micropeptides in developing cortex and cultured neurons.

Although the functional repertoire of these peptides is only beginning to emerge, current data suggest roles in synaptic vesicle recycling, cytoskeletal remodelling, calcium buffering, and modulation of local translation at dendrites—processes central to synaptic transmission. Within neurons, circRNAs integrate transcriptional and post-transcriptional control to fine-tune synaptic transmission, axonal guidance, and neuroimmune signalling. CircRNAs participate in several conserved biochemical pathways—including MAPK/ERK signalling, calcium-dependent plasticity, oxidative-stress pathways, and chromatin-modifying cascades (Table 3). Their stability, conservation, and precise subcellular localization collectively position circRNAs as fundamental components of neuronal regulatory architecture and as promising biomarkers and therapeutic targets.

### 4.2. circRNAs in Autism Spectrum Disorder

Convergent genetic, environmental, and post-transcriptional evidence identifies circRNAs as dynamic regulators in ASD. Genome-wide analyses reveal that circRNA quantitative trait loci (circQTLs) modulate circRNA expression and contribute to ASD risk by reshaping circRNA–miRNA–mRNA networks controlling synaptic and chromatin-regulatory genes [51].

Environmental perturbations also remodel circRNA expression during neurodevelopment. In a murine model of air pollution exposure, Xie et al. identified 343 differentially expressed circRNAs mapping to synaptic and oxidative stress pathways [52]. Notably, circRNAs derived from Dlgap1 and Grin2b, key postsynaptic density genes, were dysregulated—linking environmental insults to excitatory-circuit disturbances.

Mechanistically, circRNAs frequently regulate ASD pathways via competing endogenous RNA (ceRNA) networks. Plasma exosome sequencing identified 46 core circRNAs predicted to interact with ASD-relevant miRNAs such as miR-181b-5p, miR-15b-5p, and miR-218-5p [53]. These modules converge on MAPK and calcium signalling pathways, key regulators of synaptic strength and neural plasticity.

Computational tools such as CircMiMi [54] extend these networks by predicting conserved circRNA–miRNA–mRNA interactions, uncovering axes such as circ_0004104–miR-9–BCL11A, which influence neuronal differentiation. Integration across multi-omics datasets provides a scalable approach to prioritizing functional circRNAs for mechanistic study and therapeutic development. Together, circRNAs emerge as multimodal integrators linking inherited variation, environmental stress, and post-transcriptional coordination—highlighting their potential as stable biomarkers and mechanistic mediators of ASD pathology.

### 4.3. circRNA Networks Linking Developmental Injury, Plasticity, and Neuropsychiatric Pathology

Perinatal brain injury provides a natural context to examine how circRNAs regulate neural and muscular circuitry under early-life stress. Genome-wide circRNA sequencing in infants with cerebral palsy identified hsa_circ_0086354 as a differentially expressed transcript with high diagnostic accuracy, participating in inflammatory and cytoskeletal-remodelling networks [55].

In muscle satellite cells of patients with spastic cerebral palsy, circNFIX modulates MEF2C, linking myogenic and neurogenic transcriptional regulation [56]. These data suggest that circRNAs act at both neurogenic and myogenic interfaces, integrating central and peripheral transcriptional responses to developmental injury, and explaining the persistence of motor deficits after hypoxic–ischaemic events.

Epitranscriptomic regulation adds another layer of circRNA control. In APP/PS1 models, widespread m^6^A methylation of circRNAs influences synaptic transmission and innate immune responses [57]. Similarly, Siqueira et al. observed coordinated circRNA alterations with transcribed ultraconserved regions in Rett syndrome models [58], converging on chromatin and synaptic pathways regulated by MeCP2.

Early psychiatric disorders also exhibit circRNA dysregulation. In first-episode schizophrenia, region-specific modules such as hsa_circ_CORO1C–miR-708-3p–JARID2/LNPEP implicate circRNAs in chromatin modification and neurodevelopmental signalling [59]. Transcriptome-wide validation confirms overlap with dopaminergic and neuroinflammatory pathways [60]. Du et al. showed that plasma EV-derived circRNAs form ceRNA networks enriched in neuronal adhesion and oxidative-stress genes [61], indicating cross-compartment communication. Li et al. demonstrated that retroviral envelope activation (ERVWE1) induces circ_0001810, triggering mitochondrial dysfunction [62].

Beyond pathology, circRNAs shape developmental circuits. Qi et al. identified a circRtn4–miR-24-3p–CHD5 axis promoting neurite outgrowth via derepression of the chromatin remodeller CHD5 [63]. In Drosophila, the circular RNA Edis participates in an Edis–Relish–castor loop regulating neuronal differentiation and immune responses [64,65], illustrating evolutionary conservation. Glial circRNAs also contribute: circFGFR2 triggers astrocytic pyroptosis in ischaemic injury [66], revealing mechanisms shared between developmental and inflammatory contexts.

circRNAs mediate both neuroprotection and neurodegeneration. For example, hsa_circ_0000288 stabilizes Caprin1, maintains synaptic integrity in epilepsy [67]; circ_0049472 regulates PDE4A via miR-22-3p in Alzheimer’s and developmental disorders [68]. Recurrent circRNA–miRNA–mRNA axes involving MAPK, miR-22-3p, CHD5, and synaptic cytoskeletal pathways suggest a unifying model in which circRNAs function as long-lived scaffolds preserving transcriptional stability across developmental windows.

At the systems level, circRNAs convert genetic variation into post-transcriptional misregulation via circQTLs [51]; encode environmental memory, as in PM2.5-induced neurotoxicity [52]; and mediate intercellular communication through exosomal transport [53]. Mechanistically, they coordinate ceRNA crosstalk [63], chromatin modification [59], and immune–metabolic integration [64,65] (Table 3). Together, these findings define circRNAs as evolutionarily conserved regulatory nodes connecting chromatin dynamics, neuronal excitability, metabolic stress, and immune adaptation. Their detectability in biofluids, stability, and reversible regulation make circRNAs highly attractive biomarkers and therapeutic targets in neurodevelopmental medicine.

### 4.4. Cross-Disorder circRNA Dysregulation Across Neurodevelopmental Disorders

Current evidence suggests that certain circRNA-regulated pathways may be shared across multiple NDDs, although direct comparative circRNA transcriptome studies outside ASD remain limited. High-resolution cortical transcriptomics in ASD has identified over 60 dysregulated circRNAs and thousands of circRNA–miRNA–mRNA regulatory axes enriched in synaptic organization, chromatin regulation, and inhibitory–excitatory balance. Several ASD-associated circRNAs, such as circARID1A, modulate miR-204-3p and downstream neurodevelopmental genes including NLGN1, STAG1, and UBA6. Notably, UBA6 deficiency is linked to intellectual disability phenotypes, suggesting that circRNA-mediated regulation of this axis may span multiple NDD categories. Although dedicated circRNA profiling in ADHD or idiopathic intellectual disability has not yet been reported, converging pathway-level evidence indicates potential overlap. Many circRNA-regulated modules in ASD, such as MAPK/ERK signalling, Notch-dependent neurogenesis, GABAergic synaptic pathways, and oxidative–metabolic stress cascades, are also implicated genetically and functionally in ADHD and ID. Current data reveal pathway-level convergence rather than disorder-specific circRNA signatures. ASD provides the strongest empirical evidence, while cross-disorder overlap is suggested indirectly through shared gene targets, common synaptic and chromatin pathways, and syndromic ID models. Systematic circRNA profiling in ADHD and ID will be essential to determine whether disease-specific or pan-NDD circRNA modules truly exist.

CircRNAs present compelling mechanistic potential due to their stability, evolutionary conservation, and modular ceRNA architectures. However, circRNA function in mammalian neurons remains much less experimentally established than suggested by associative studies. Evidence for circRNA-derived peptides, activity-dependent circRNA regulation, and circRNA-controlled chromatin programs is promising but incomplete. A key challenge moving forward is differentiating circRNAs that act as true regulatory nodes from those reflecting transcriptional noise or host-gene expression. Combining long-read sequencing, ribosome profiling, and perturbation tools such as CIRI-CRISPR will allow the field to resolve the mechanistic relevance of circRNAs in neurodevelopmental disorders.

## 5. tsRNAs and piRNAs as Emerging Neuroregulators

Transfer-RNA-derived small RNAs (tsRNAs) and PIWI-interacting RNAs (piRNAs) have moved from the periphery of RNA biology to the centre of neural regulation. Once viewed as germline-restricted or stress-responsive species, both classes are now recognised as integral components of the neuronal transcriptome that couple chromatin control, translational tuning, and epigenetic memory to developmental trajectories and plasticity.

Their distinct biogenesis pathways, dense modification landscapes, and persistence in post-mitotic neurons provide a molecular substrate for long-term information storage, linking metabolic and environmental cues to durable changes in neural circuits. Mechanistically, tsRNAs and piRNAs regulate neurodevelopment through three main axes: (1) translational and ribosomal control, (2) chromatin and genome stability, and (3) intercellular or intergenerational information transfer (Table 4).

### 5.1. Biogenesis and Functional Logic of tsRNAs

tsRNAs arise from precise cleavage of precursor or mature tRNAs by Dicer, ELAC2/RNase Z, RNase P, or angiogenin, producing tRF-5, tRF-3, tRF-1, internal tRFs (i-tRFs), and longer stress-induced halves (tiRNAs). Their abundance and sequence composition are shaped by tRNA modification density, aminoacylation state, and cell-type specificity [69,78]. Under oxidative or nutrient stress, angiogenin generates 5′-tiRNAs that inhibit cap-dependent translation by displacing the eIF4F complex, redirecting energy toward survival programmes. Loss of 5-methylcytosine (m^5^C, via NSUN2/DNMT2) destabilises neuronal tRNAs, increasing tsRNA production and impairing differentiation; N^1^-methyladenosine (m^1^A) and 7-methylguanosine (m^7^G) tune AGO loading and codon-specific translation, connecting tsRNAs to MAPK/mTOR and neurotrophin signalling [69,71,72]. Beyond global repression, tsRNAs execute sequence-specific, AGO-dependent silencing that regulates neurotrophin, MAPK, and mTOR pathways—key regulators of dendritic growth and plasticity [71].

In Alzheimer’s-related injury, the 5′-tRF tRFAla-AGC-3-M8 attenuates microglial activation and neuronal apoptosis by repressing the EphA7–ERK1/2–p70S6K axis [79]. A striking dimension of tsRNA biology is their participation in intergenerational inheritance. Ageing reshapes sperm tsRNA repertoires targeting synaptic and neurogenic pathways; microinjection of aged-sperm tsRNAs into naïve zygotes reproduces anxiety-like behaviours and forebrain transcriptional changes in offspring [72]. Therapeutic strategies include restoring tRNA-modification balance, delivering synthetic neuroprotective tsRNAs, or inhibiting pathogenic fragments with antisense oligonucleotides [73].

### 5.2. piRNAs in Neural Regulation and Neurodevelopmental Disorders

piRNAs are generated through a Dicer-independent pathway involving Zucchini cleavage and HEN1-mediated 3′ methylation. They bind PIWI proteins (PIWIL1/HIWI, PIWIL2/HILI) and canonically enforce transposon silencing via the ping-pong cycle. In neurons, however, piRNAs extend far beyond genome defence, regulating mRNA stability, alternative splicing, synaptic translation, and chromatin accessibility. Disrupting PIWIL1/PIWIL2 impairs dendritic spine morphology and long-term potentiation, establishing a causal role in cognition and memory [74].

Although classically studied in neurodegeneration, piRNA dysregulation converges on pathways shared with NDDs. In Parkinson’s disease, altered piRNA/PIWI expression correlates with α-synuclein pathology in human tissue and *C. elegans* models [80,81]. Loss of tdp-1 further disrupts piRNA maturation and exacerbates neurotoxicity [82]. In Alzheimer’s disease, plasma piRNA profiling reveals hundreds of dysregulated piRNAs regulating amyloid processing, tau phosphorylation, and neuroinflammation [83,84,85].

In ASD and other childhood NDDs, small RNA sequencing of plasma and faecal samples identifies brain-enriched piRNAs linked to synaptic, immune, and mitochondrial pathways [75,76,77]. Mechanistically, nuclear piRNAs can recruit DNMTs, HMTs (e.g., SETDB1) to synaptic-gene promoters, enforcing long-term gene silencing [86]. Cytoplasmic piRNAs bind long 3′-UTRs to tune local translation at synapses. Together, these pathways place piRNAs at the intersection of epigenetic memory, synaptic scaling, and excitatory–inhibitory homeostasis in NDDs.

### 5.3. Interconnected Small-RNA Networks Linking Neurodevelopment

An emerging dimension of small-RNA biology lies in the microbiome–piRNA/tsRNA axis. In ASD cohorts, coordinated shifts in microbiota composition and host piRNA expression have been detected in stool and serum [76]. Bacterial-derived tRNA fragments can enter host circulation and modulate immune pathways, influencing neural development. Moreover, bacterial-derived tRNA fragments can enter the host circulation and modulate immune pathways that feed back onto neural development. Integrative analyses combining metagenomic, small-RNA, and metabolomic data are expected to uncover a multidirectional communication network in which tsRNAs and piRNAs translate microbial and environmental signals into epigenetic and neurodevelopmental outcomes. Viewed together, tsRNAs and piRNAs define a unified regulatory axis that complements and extends the established roles of miRNAs, lncRNAs, and circRNAs. Both classes link environmental and metabolic stimuli to transcriptional and translational control through chemically diverse, highly stable molecules capable of acting across cellular and temporal scales.

Enzymes governing tRNA methylation (NSUN2, TRMT6/61, METTL1) and piRNA maturation (HEN1, Zucchini) couple RNA modification to chromatin remodelling. Loss-of-function mutations in these pathways produce overlapping phenotypes—microcephaly, intellectual disability, and autistic-like behaviour—highlighting shared epigenetic control of neural development [70,74]. The remarkable stability and vesicular packaging of tsRNAs and piRNAs enable their transfer between cells and even across generations. In both extracellular vesicles and gametes, they transmit environmental or ageing-related information that can shape offspring neurodevelopment and stress resilience [72,75].

The presence of tsRNAs and piRNAs in plasma, cerebrospinal fluid, and faeces makes them minimally invasive indicators capable of distinguishing disease states and progression [76,83]. Emerging RNA-based therapeutics—including antisense oligonucleotides, CRISPR-mediated modulation, and synthetic mimetics—seek to restore physiological small-RNA signalling and normalise neural gene expression [79]. Together, these interconnected systems reveal a previously unrecognised layer of neurogenetic regulation that bridges metabolism, environment, and inheritance. tsRNAs act as rapid translators of metabolic and immune signals into local translational programmes, whereas piRNAs enforce durable epigenetic memory through PIWI-guided chromatin silencing. Their cooperation establishes a molecular grammar for encoding life-history information into neuronal function. As modification-aware single-cell and spatial transcriptomic technologies mature, resolving the cell-type-specific expression and interaction of tsRNAs and piRNAs will illuminate how these unconventional ncRNAs sculpt neural development, plasticity, and disease. This emerging paradigm recasts tsRNAs and piRNAs not as auxiliary regulators, but as foundational architects of the RNA-based regulatory circuitry that underpins cognition and neurodevelopmental health (Table 4).

Compared with miRNAs and lncRNAs, tsRNAs and piRNAs represent the most conceptually novel yet least mechanistically resolved ncRNA classes in the brain. Their dual roles in translation, stress adaptation, and epigenetic memory raise the possibility that they encode long-term developmental information. However, whether these small RNAs directly regulate neuronal differentiation and circuit formation, or instead reflect upstream metabolic and chromatin states, is still unclear. Establishing causality will require integrating enzymatic perturbations, RNA modification profiling, and intergenerational transmission models. These RNA classes may ultimately reveal mechanisms that bridge metabolism, environment, and heritable neurodevelopmental vulnerability.

## 6. ncRNAs as Biomarkers: From Brain to Blood

The recognition that ncRNAs circulate beyond the confines of the central nervous system has transformed the search for molecular biomarkers of NDDs. Unlike protein markers, which often degrade rapidly or reflect downstream pathology, ncRNAs display exceptional biochemical stability due to compact secondary structures, association with RNA-binding proteins, and encapsulation within extracellular vesicles (EVs) [1,2]. These structural safeguards allow diverse ncRNA species, including miRNAs, lncRNAs, circRNAs, tsRNAs, piRNAs, and snoRNAs to persist in plasma, saliva, cerebrospinal fluid (CSF), and urine. Of particular relevance to neurodevelopment, these circulating ncRNAs frequently mirror processes such as synaptic maturation, metabolic stress response, neuroimmune activation, and axonal plasticity—mechanisms that originate during early brain development and continue across the lifespan.

Across ASD and ADHD, peripheral transcriptomic analyses have identified reproducible ncRNA signatures paralleling the synaptic, mitochondrial, and immune gene networks disrupted in cortical and striatal regions. For example, serum miR-195-5p is elevated in ASD and correlates with clinical severity through FGFR1 regulation [7], whereas miR-499a-5p demonstrates high diagnostic accuracy and associates with amygdala–caudate morphology [8]. lncRNAs show comparable diagnostic potential: NEAT1, a regulator of UBE3A transcription and neuroinflammation [34], and H19, which modulates oxidative-stress pathways via miR-484 [38], are consistently dysregulated in ASD cohorts. Mounting evidence indicates that EVs are the principal vehicles transporting brain-derived ncRNAs into circulation. Neuron- and astrocyte-derived exosomes can cross the blood–brain barrier while preserving their RNA cargo.

Beyond EV-mediated transcytosis, several additional physiological routes enable CNS-derived ncRNAs to enter the peripheral circulation. The glymphatic system clears extracellular RNA-containing vesicles and ribonucleoprotein complexes from the interstitial space into cerebrospinal fluid (CSF), which subsequently drains into meningeal lymphatic vessels and cervical lymph nodes before reaching the bloodstream. Perivascular pathways likewise transport RNA cargo from the brain parenchyma into systemic circulation, particularly during sleep- and activity-dependent fluid exchange. Importantly, circulating ncRNAs do not exclusively originate from the CNS: immune cells, endothelial cells, hepatocytes, and adipose tissues also release abundant ncRNA species into plasma, necessitating careful interpretation when assigning biomarker specificity to neural processes. The remarkable stability of circulating ncRNAs is explained by several biochemical safeguards. Encapsulation within EV lipid bilayers protects RNA molecules from RNase digestion and preserves their secondary structure during transport. Independent of vesicular packaging, many miRNAs, tsRNAs, and piRNAs circulate bound to RNA-binding proteins such as AGO2, YBX1, and nucleophosmin, while chemical modifications, including 2′-O-methylation, m^7^G, m^6^A, and m^5^C, further enhance nuclease resistance. These combined protective mechanisms maintain ncRNA integrity over prolonged periods, enabling their detection in biofluids and supporting their utility as robust, temporally stable biomarkers of neurodevelopmental physiology and pathology.

Plasma EV profiling reveals enrichment for synaptic miRNAs and circRNAs involved in axon guidance and plasticity [6,22,53]. These EV-associated ncRNAs likely originate from activity-dependent secretion, coupling neuronal firing and glial signalling to systemic circulation. Their encapsulation protects them from degradation and confers exceptional reproducibility, making EV-bound ncRNAs superior to freely circulating species for biomarker discovery.

Recent high-throughput studies extend this paradigm to additional ncRNA classes. piRNAs, once considered germline-restricted, are now detected in both brain and plasma, with disease-specific signatures. Large-scale sequencing identified hundreds of brain-enriched piRNAs distinguishing NDDs and neurodegenerative diseases such as Alzheimer’s disease (AD) with high accuracy [83,84,85]. These piRNAs converge on chromatin-regulatory and synaptic pathways, suggesting that neuronal epigenetic programmes are echoed in circulation. Similarly, tRNA-derived fragments (tRFs) generated by angiogenin or Dicer regulate translation and oxidative-stress responses; altered methylation and abundance in patient serum mark metabolic imbalance [69]. In both AD and ASD cohorts, tRFs associated with oxidative phosphorylation show consistent dysregulation, implicating tRNA-modifying enzymes such as NSUN2 and TRMT6/61 in neural vulnerability [2,70]. Previously overlooked ncRNAs—including snoRNAs and Y RNAs—are also emerging as circulating indicators. C/D box snoRNAs SNORD115 and SNORD116, transcribed from the imprinted 15q11–13 locus, are enriched in plasma EVs of AD patients and distinguish them from controls with high accuracy. Comparable snoRNA and Y-RNA fragments have been detected in ASD plasma [76], suggesting evolutionarily conserved release mechanisms and cross-disease biomarker potential.

Realizing the clinical promise of ncRNA biomarkers requires standardized analytical frameworks. Pre-analytical variables—sample collection, haemolysis assessment, and data normalization—must be rigorously controlled [1]. Comparative studies demonstrate that integrating total plasma RNA with EV-enriched RNA profiles enhances reproducibility and signal-to-noise ratio. Advanced detection platforms, including small-RNA-seq, total-RNA-seq, EV-RNA-seq, and targeted RT-qPCR panels, now permit comprehensive quantification across ncRNA classes. Analytically, machine-learning classifiers trained on multi-class ncRNA datasets outperform single-class models. In AD, combined lncRNA–piRNA–snoRNA signatures achieved AUC ≈ 0.96 [83,84], a framework now being extended to NDD subtype stratification and longitudinal monitoring.

Collectively, circulating ncRNAs constitute a stable, information-rich interface between the brain and periphery. Their biochemical stability [1,2], cell-type specificity [6,22], and cross-class complementarity define a multilayered communication network linking neural metabolism, epigenetic state, and systemic physiology. As modification-aware single-cell and spatial transcriptomic technologies evolve, it will become possible to map ncRNA release, trafficking, and uptake across neural circuits in vivo. Such frameworks promise blood-based monitoring of neurodevelopment and synaptic plasticity, transforming ncRNAs from static diagnostic markers into dynamic sentinels of brain health. Ultimately, circulating ncRNAs may serve not merely as biomarkers but as integrative molecular reporters—bridging the molecular, cellular, and systemic dimensions of neurodevelopmental biology.

Although circulating ncRNAs show great diagnostic promise, the mechanistic interpretation of their origin and specificity remains incomplete. Distinguishing CNS-derived RNA signals from contributions of peripheral immune, endothelial, or metabolic tissues is a major challenge. Moreover, the stability and heterogeneity of extracellular vesicle cargo introduce additional analytical complexity. Future biomarker frameworks must integrate EV profiling, cell-type-specific RNA release signatures, and longitudinal sampling to identify robust, mechanistically interpretable biomarkers for neurodevelopmental disorders (Table 5).

## 7. Therapeutic Targeting of ncRNAs: From Bench to Bedside

The advent of ncRNA therapeutics marks a conceptual shift from single-gene correction toward reprogramming entire regulatory networks. Because ncRNAs coordinate chromatin architecture, translational control, and synaptic plasticity, interventions at this level offer the ability to recalibrate dysregulated circuits that drive NDDs. Rather than editing individual loci, ncRNA-based strategies aim to restore network equilibrium, achieving molecular homeostasis through systemic regulatory rewiring (Table 6).

Preclinical studies have demonstrated that re-establishing ncRNA balance can reverse both behavioural and molecular abnormalities in NDD models. In ASD, lentiviral re-expression of miR-137 or miR-215-5p restores glucose metabolism, suppresses neuroinflammation, and improves social behaviour [6,22]. Conversely, antisense inhibition of NEAT1, a lncRNA that recruits YY1 to the UBE3A promoter, mitigates valproic-acid-induced synaptic dysfunction [34]. In Angelman syndrome, antisense oligonucleotides (ASOs) targeting the UBE3A-ATS transcript unsilence the normally repressed paternal UBE3A allele—representing a landmark example of epigenetic reactivation through ncRNA modulation [46]. These findings underscore ncRNAs as master regulators capable of reinstating transcriptional and metabolic stability when therapeutically tuned.

Emerging work extends ncRNA intervention to other molecular classes. Manipulating circRNAs via siRNA-mediated knockdown of pathogenic isoforms or overexpression of neuroprotective ones can remodel synaptic gene networks and attenuate neuroinflammation. Likewise, tsRNAs can be indirectly modulated by restoring the activity of tRNA-modifying enzymes such as NSUN2 or TRMT6/61, re-establishing the balance between translation repression and neuronal differentiation [70]. Therapeutic exploration of piRNAs is at an earlier stage, yet enhancement of PIWI-protein activity or supplementation with protective piRNA pools may suppress transposon reactivation and neuroinflammatory cascades shared mechanisms in ageing and neurodegeneration. Together, these approaches highlight a continuum of regulatory intervention, where ncRNAs serve as molecular levers bridging chromatin state, translation efficiency, and cellular stress resilience.

The primary obstacle to ncRNA therapeutics remains efficient, cell-specific delivery across the blood–brain barrier (BBB). Advances in vector engineering now enable unprecedented precision. Adeno-associated virus (AAV) vectors and lipid nanoparticles (LNPs) permit region- or cell-type-selective expression of ncRNA constructs, while exosome-based delivery offers a biologically compatible and immunologically inert alternative. In perinatal brain injury, exosomes derived from bone-marrow mesenchymal stem cells transfer neuroprotective miRNAs that rescue cortical neurons and reduce gliosis [30]. Similarly, in Rett syndrome, manipulation of the X chromosome, linked lncRNA pathways via engineered vesicles, ameliorates neurological deficits [29].

Integration of CRISPR–dCas9 systems with ncRNA promoters enables locus-specific activation or repression of endogenous genes, uniting RNA therapeutics with programmable epigenetic editing [46]. These innovations collectively chart a path toward spatiotemporally controlled ncRNA therapeutics capable of modulating neural networks with surgical precision.

Despite these advances, several hurdles remain. Off-target hybridization, activation of innate immune sensors, and prolonged intracellular persistence demand rigorous pharmacological oversight. Comprehensive off-target mapping, immunogenicity testing, and pharmacokinetic modelling are essential to ensure safety. Next-generation designs incorporating switchable, biodegradable, or ligand-targeted systems aim to minimize immune activation while preserving delivery fidelity. Looking ahead, ncRNA pharmacology stands at the threshold of clinical translation. The convergence of synthetic biology, RNA chemistry, and neuro-epigenetics promises an era in which fine-tuning ncRNA circuitry may restore neurodevelopmental stability not by silencing disease, but by rebalancing the symphony of the neural transcriptome.

## 8. Cell-Type-Specific ncRNA Networks Revealed by Single-Cell and Spatial Omics

The cellular heterogeneity of the human brain has long obscured the mechanistic understanding of ncRNA function in NDDs. Recent advances in single-cell and spatial multi-omics now enable ncRNA expression to be mapped with unprecedented resolution—across cell types, developmental stages, and anatomical domains—revealing how discrete ncRNA programs orchestrate circuit formation and confer selective vulnerability to disease [33].

Single-nucleus RNA sequencing of ASD cortex demonstrates that excitatory projection neurons, interneurons, and astrocytes possess distinct lncRNA and circRNA repertoires, many co-regulated with synaptic and immune pathways [36,37]. Among these, NEAT1 and MEG3 are selectively upregulated in astrocytes, where they coordinate inflammatory and stress-response gene programs [34], whereas neuronal-specific lncRNAs interact with chromatin remodellers to govern activity-dependent transcription [32]. Integration of single-cell transcriptomes with chromatin-accessibility and proteomic datasets identifies ncRNA-driven regulatory modules that define lineage-specific sensitivity to developmental perturbations, offering a molecular rationale for the cell-type-restricted pathology characteristic of NDDs.

Spatial transcriptomics extends these insights by preserving anatomical context. In both human and model systems, circRNAs enriched in cortical layer V neurons and hippocampal pyramidal cells localize to loci involved in synaptic-vesicle cycling and structural plasticity [58]. Conversely, microglial lncRNAs cluster within neuroimmune hubs of the prefrontal cortex, aligning with region-specific inflammatory circuits [32]. These spatially constrained transcriptional architectures suggest that ncRNA dysregulation produces circuit-specific pathophysiology rather than uniform global dysfunction, an emerging hallmark of complex neurodevelopmental disease.

Although most single-cell RNA-sequencing platforms enrich for polyadenylated transcripts and therefore capture circRNAs only sparsely, emerging poly(A)-independent technologies—including SMARTer total-RNA single-cell sequencing, Nanopore-based long-read profiling, and CIRI-long—are beginning to resolve circRNA expression with true cell-type and laminar specificity. These methods reveal that synaptic circRNAs are highly enriched in projection neurons and inhibitory interneurons, mirroring the cell populations most consistently altered in ASD. Spatial transcriptomic approaches further highlight the potential of circRNAs to explain circuit-level dysfunction in ASD. CircRNAs implicated in ASD, such as circARID1A or circRtn4, participate in pathways critical for dendritic growth, axonal projection, and excitatory–inhibitory balance. These processes are known to be layer-specific within corticostriatal and corticothalamic circuits. As higher-resolution platforms such as Slide-seqV2, Stereo-seq, and long-read spatial transcriptomics continue to evolve, they are expected to uncover layer-dependent circRNA expression signatures and cell-type-specific ceRNA networks that better explain ASD-associated connectivity deficits.

Recent total-RNA single-cell technologies, capable of capturing non-polyadenylated species, have expanded ncRNA profiling to tsRNAs and piRNAs, providing the first cell-resolved view of small-RNA biology in the developing brain. These datasets reveal developmentally staged waves of small-RNA activity: tsRNAs dominate early neurogenesis, linking metabolic state to translational control, whereas piRNAs emerge during synaptogenesis, maintaining chromatin stability and repressing transposon activity [69,74]. Integration with live-cell imaging and metabolic labelling now enables real-time visualization of ncRNA turnover, illuminating how local RNA metabolism coordinates dendritic remodelling and synaptic plasticity.

As computational frameworks mature, the convergence of single-cell, spatial, and epitranscriptomic data is poised to generate three-dimensional atlases of ncRNA regulation across developmental time and brain architecture. These integrative maps will link molecular heterogeneity to neural-circuit function, forming the empirical basis for precision diagnostics and cell-type-specific RNA therapeutics. Ultimately, this emerging framework redefines ncRNAs not as static transcripts but as dynamic regulatory codes, encoding spatial and temporal logic into the developmental blueprint of the human brain.

Single-cell and spatial omics have begun to reveal fundamental organizing principles of ncRNA networks, yet the field is far from defining a unified regulatory atlas. Current datasets highlight a hybrid model in which universal motifs, ceRNA loops, RNA–RBP hubs, and chromatin–ncRNA feedback are deployed within highly context-specific architectures defined by cell identity and circuit organization. A key future direction will be integrating perturbation-based single-cell transcriptomics with long-read and epitranscriptomic methods to map hierarchical ncRNA regulation with true cell-type specificity.

### Future Directions and Challenges

Collective evidence now positions ncRNAs as the molecular infrastructure linking the genome, environment, and neuronal identity. Yet despite remarkable advances, major conceptual and translational challenges must be overcome before ncRNA biology can be fully translated into clinical neuroscience. Mechanistically, ncRNAs function not as isolated regulators but as interdependent components of a multilayered post-transcriptional ecosystem. miRNAs, lncRNAs, circRNAs, tsRNAs, and piRNAs share overlapping targets and RNA-binding partners, forming dynamic feedback circuits that fine-tune neuronal gene expression [32,78]. Disentangling this regulatory crosstalk is crucial for understanding emergent transcriptomic behaviours. Next-generation computational frameworks that integrate sequence complementarity, secondary structure, and spatial co-localization will be instrumental in predicting ncRNA–RNA and ncRNA–protein interactions at a systems level. Advances in machine learning and network biology are beginning to reveal how perturbations in one ncRNA class can ripple through the broader regulatory architecture, generating complex and sometimes nonlinear phenotypic outcomes.

An additional layer of regulation arises from the epitranscriptomic code—chemical modifications such as 5-methylcytosine (m^5^C), N^6^-methyladenosine (m^6^A), and pseudouridine (Ψ)—that diversify RNA stability, localization, and translation potential. Cell-type-resolved mapping of these marks will clarify how chemical diversity translates into functional specialization during brain development and plasticity. Understanding how ncRNAs intersect with RNA modifications to modulate chromatin state, neuronal differentiation, and circuit homeostasis represents one of the most pressing frontiers in neuro-epigenetics.

Translationally, the field now faces the challenge of standardization and reproducibility. Large-scale, multi-centre cohorts with harmonized biofluid processing, unified normalization pipelines, and deep phenotypic characterization are required to validate ncRNA biomarkers across populations and developmental stages. Integrating ncRNA profiles with proteomic, metabolomic, and neuroimaging data will be essential to achieve the multidimensional precision necessary for regulatory qualification of diagnostic panels [75,83]. Establishing global data-sharing frameworks and analytical standards will accelerate reproducibility and foster cross-cohort meta-analysis, transforming ncRNA biomarker research from exploratory discovery to clinical validation.

On the therapeutic front, delivery and safety remain the key constraints. The convergence of AAV vectors, LNPs, and exosome-based carriers offers promising strategies to cross the blood–brain barrier and achieve cell-type-specific RNA delivery [29,30]. In parallel, CRISPR–dCas9 epigenetic editing now enables locus-specific activation or repression of ncRNA genes without inducing double-strand DNA breaks, allowing durable correction of transcriptomic dysregulation [46]. Together, these advances define a rapidly maturing toolkit for precision RNA therapeutics in neurodevelopmental medicine.

Although ncRNA repertoires vary markedly across neuronal and glial cell types, emerging single-cell and spatial datasets suggest that their regulatory networks follow several conserved organizational principles. Recurrent motifs, such as ceRNA competition loops, RNA–RBP hub structures, and chromatin–ncRNA feedback circuits, reappear in cortical, hippocampal, and cerebellar cell populations, indicating a shared regulatory grammar. However, the specific topology of these networks is strongly shaped by cell identity, developmental timing, and regional specialization, producing distinct ncRNA modules in excitatory projection neurons, inhibitory interneurons, astrocytes, and microglia. Thus, ncRNA regulation operates through universal principles deployed in context-specific architectures, a duality that helps explain the selective circuit vulnerability observed in neurodevelopmental disorders.

The next frontier lies in connecting ncRNA dynamics to developmental time. Integration of spatial-omics atlases with longitudinal clinical and genetic datasets will enable reconstruction of ncRNA expression trajectories from embryogenesis through adulthood [33]. Such frameworks will illuminate how early transcriptomic perturbations propagate through brain maturation, linking molecular alterations to circuit architecture and behavioural phenotype. Ultimately, this systems-level perspective will enable predictive modelling of neurodevelopmental trajectories, bridging genotype, cell-type vulnerability, and cognitive outcome.

## 9. Conclusions

NDDs arise from disruptions of tightly coordinated molecular networks that govern neurogenesis, synaptogenesis, and circuit maturation. Across these regulatory layers, ncRNAs have emerged as central integrators of chromatin state, transcriptional programs, translational control, and intercellular communication. Despite rapid methodological advances, from multi-omics profiling to single-cell and spatial transcriptomics, our mechanistic understanding of ncRNA function across distinct developmental windows and cell types remains incomplete.

First, for many ncRNA subtypes, the causal direction between dysregulation and NDD pathology remains unclear; most functional data come from rodent or in vitro systems, with limited human validation. Second, large discrepancies persist among published datasets due to small sample sizes, heterogeneous clinical cohorts, and inconsistent analytical frameworks. Third, ncRNA regulatory networks are highly cell-type- and region-specific, yet most studies rely on bulk tissue, obscuring circuit-level mechanisms relevant to ASD, ADHD, and intellectual disability. Fourth, the mechanisms by which ncRNAs travel from the CNS to peripheral circulation, and the extent to which peripheral signals faithfully reflect brain states, require systematic clarification.

The accumulating evidence highlights several promising directions. Circulating ncRNAs, particularly those encapsulated in extracellular vesicles, show strong potential for early diagnosis, subtype stratification, and monitoring of treatment response. Integrating multi-class ncRNA signatures with machine-learning models may transform screening and risk prediction in NDDs. Antisense oligonucleotides, miRNA mimics, circRNA overexpression constructs, and CRISPR-based epigenetic editing represent an expanding therapeutic toolkit. Mapping ncRNA regulatory networks across single-cell and spatial atlases will enable the development of interventions tailored to vulnerable cell types, such as cortical projection neurons, inhibitory interneurons, or astrocytic immune hubs in ASD. Longitudinal, multi-omics datasets are poised to link developmental trajectories of ncRNA expression with behavioural outcomes, enabling predictive models that guide individualized therapies.

To realize the full potential of ncRNA-based diagnostics and therapeutics, several priorities must be addressed: Large, harmonized cohorts integrating genomics, epitranscriptomics, and clinical phenotyping; Single-cell total-RNA platforms capable of capturing miRNAs, lncRNAs, circRNAs, tsRNAs, and piRNAs simultaneously; partially resolved ncRNA atlases across human cortical layers and subcortical circuits; In vivo perturbation systems for causal testing of ncRNA networks; Delivery technologies enabling targeted modulation of ncRNAs in defined neuronal subtypes; Long-term safety studies evaluating immunogenicity, off-target effects, and developmental plasticity.

In summary, ncRNAs constitute a multilayered regulatory architecture linking genome regulation, environmental responsiveness, and neural circuit development. By integrating mechanistic studies with emerging single-cell and spatial transcriptomic technologies, future research will fill key knowledge gaps and accelerate the development of precise, mechanism-based ncRNA therapeutics for neurodevelopmental disorders.

## Figures and Tables

**Table 1:** Integrated evidence of key miRNAs in neurodevelopmental disorders.

miRNA	Pathway	Disease	Study Type	References
miR-181a-5p	Mitochondrial OXPHOS repression; metabolic stress	ASD	Post-mortem cortex	[4]
miR-34a	Mitochondrial dysfunction; MAPK modulation	ASD	Large-cohort transcriptomics	[4]
miR-23b/27b/24 cluster	Epigenetic and intergenerational effects	ASD (maternal hypoxia model)	Mouse litters	[5]
miR-215-5p	NEAT1/MAPK1/CRMP2 synaptic pathway	ASD (VPA model)	Rodent model	[6]
miR-195-5p	FGFR1 neurodevelopmental pathways	ASD	Human plasma	[7]
miR-499a-5p	Structural changes (amygdala–caudate)	ASD	Multi-cohort study	[8]
miR-21	Vascular stress, neuroimmune signalling	ASD	Systems biology analysis	[9]
miR-146a/miR-181/miR-21	Chromatin & epigenetic regulation	ASD	Computational/tissue-level evidence	[10]
miR-34c-3p/miR-138-1	BDNF–TrkB signalling	ADHD	Case–control	[11]
miR-130	SNAP-25 presynaptic machinery	ADHD/lead exposure	Rodent model	[12]
miR-26b-5p/miR-185-5p/miR-142-3p	Dopaminergic/synaptic regulation	ADHD	Multi-cohort clinical studies	[13,14,15]
miR-9/miR-124/miR-132	Neural progenitor regulation (Notch/CHD8 axis); dendritic plasticity	General neurodevelopment	Multi-species evidence	[2,3]
miR-483-5p/miR-138-5p	Astrocyte-to-neuron EV communication	NDD spectrum	hiPSC-derived astrocyte/EV studies	[16]
miR-34a-5p	SIRT1-mediated ferroptosis	Epilepsy/injury-related neurodevelopmental deficits	Mouse models	[17]

ASD, autism spectrum disorder; ADHD, attention-deficit/hyperactivity disorder; NDD, neurodevelopmental disorder; EV, extracellular vesicle; hiPSC, human induced pluripotent stem cell; XPHOS, oxidative phosphorylation; MAPK, mitogen-activated protein kinase; CRMP2, collapsin response mediator protein 2; FGFR1, fibroblast growth factor receptor 1; BDNF, brain-derived neurotrophic factor; TrkB, tropomyosin receptor kinase B; SNAP-25, synaptosomal-associated protein 25; CHD8, chromodomain helicase DNA-binding protein 8; SIRT1, sirtuin 1.

**Table 2:** Summary of key lncRNAs implicated in neurodevelopmental disorders.

lncRNA	Pathway	Disease	Study Type	References
NEAT1	YY1 recruitment-UBE3A upregulation; neuroinflammation; oxidative stress	ASD (VPA model)	Rodent study	[34]
NEAT1–miR-215-5p–MAPK1/CRMP2 axis	Synaptic plasticity impairment; cytoskeletal destabilization	ASD	Rodent/EV studies	[6]
PCAT-29, LINC-PINT, lincRNA-p21, lincRNA-ROR, PCAT-1	Immune regulation; apoptosis; stress response	ASD	Peripheral blood, small cohorts	[35]
LINC00662, LINC00507, LINC02259	Synaptic/immune-network modulation	ASD (plasma)	Clinical cohort	[36]
SOX2-OT	Neurogenesis; glial–neuronal regulatory hub	ASD	snRNA-seq from human cortex; computational modelling	[37]
MIR155HG	Immune–synaptic crosstalk	ASD	Human snRNA-seq networks	[37]
H19/miR-484 axis	Mitochondrial stress; oxidative regulation	ASD	Human clinical study/mechanistic modelling	[38]
SINEUP–CHD8 axis	Translational enhancement without changing mRNA levels	ASD (CHD8 LOF models)	iPSC-derived neurons/mouse validation	[39]
Csnk1a1p	Kinase-related neurodevelopment signalling	ASD	GWAS/association datasets	[40]
HULC	Circadian rhythm dysregulation	ADHD	Human plasma	[41]
UCA1	Clock–metabolic coupling	ADHD	Human peripheral expression	[41]
lncMALAT1–miR-141-3p/200a-3p–NRXN1 axis	Synaptic-adhesion regulation; learning & memory	ADHD	Rodent/cell-based studies	[26]
LINC02714–SHANK2 disruption	Structural variation affecting synaptic scaffolding	ADHD	Single-case structural variant study	[42]
RNF219-AS1	White-matter microstructure, behavioural correlation	ADHD	Genetic association	[43]
BC200	Local translational control in neurons	ADHD/psychiatric disorders	SNP-association datasets	[44]
CHASERR	cis-repression of CHD2; gene-dosage regulation	CHD2-related neurodevelopmental syndrome	Human clinical cases/animal modelling	[45]
UBE3A-ATS	Paternal allele silencing via antisense transcription	Angelman syndrome	CRISPRi AAV mouse model	[46]
SPA (FUBP1-regulated)	Splicing control during neuronal differentiation	Neurodevelopmental syndromes	In vitro mechanistic studies	[47]
MNK–SYNGAP1 axis	Synaptic signalling & cognitive function	Rare NDD cases	Molecular & cellular studies	[48]

ASD, autism spectrum disorder; ADHD, attention-deficit/hyperactivity disorder; NDD, neurodevelopmental disorder; snRNA-seq, single-nucleus RNA sequencing; VPA, valproic acid; EV, extracellular vesicle; iPSC, induced pluripotent stem cell; LOF, loss of function; GWAS, genome-wide association study; SNP, single-nucleotide polymorphism; CHD8, chromodomain helicase DNA-binding protein 8; CHD2, chromodomain helicase DNA-binding protein 2; CRISPRi, CRISPR interference; AAV, adeno-associated virus; MAPK1, mitogen-activated protein kinase; CRMP2, collapsin response mediator protein 2; NRXN1, neurexin 1; SYNGAP1, synaptic Ras GTPase-activating protein 1; UBE3A-ATS, UBE3A antisense transcript; FUBP1, far-upstream element-binding protein 1.

**Table 3:** Summary of key circRNAs implicated in neurodevelopmental and early-onset neuropsychiatric disorders.

circRNA	Pathway	Disease	Study Type	References
Global circQTL–circRNA networks	Genetic variants modulate circRNA biogenesis; reshape circRNA–miRNA–mRNA networks controlling synaptic and chromatin genes	ASD	Human post-mortem brain; large-scale circQTL analysis	[51]
Air pollution-induced circRNAs (incl. circ_Dlgap1, circ_Grin2b)	Synaptic and oxidative-stress pathway dysregulation	Neurodevelopmental risk under environmental exposure	Mouse PM2.5 model; 343 DE circRNAs	[52]
Plasma exosomal circRNA panel (46 core circRNAs)	ceRNA networks involving miR-181b-5p, miR-15b-5p, miR-218-5p; MAPK & calcium signalling	ASD	Human plasma exosome sequencing; small–moderate cohorts	[53]
circ_0004104–miR-9–BCL11A axis	Regulation of neuronal differentiation; transcriptional control via BCL11A	Neurodevelopmental context (computationally inferred)	In silico prediction (CircMiMi)/literature-based support	[54]
hsa_circ_0086354	Inflammatory and cytoskeletal-remodelling networks	Cerebral palsy (perinatal brain injury)	Infant cohorts; circRNA-seq in CP	[55]
circNFIX–MEF2C axis	Regulation of MEF2C; myogenic and synaptic maturation programmes	Spastic cerebral palsy; neuromuscular interface	Human muscle satellite cells from CP patients	[56]
Globally m^6^A-methylated circRNAs	Epitranscriptomic modulation of circRNA stability, localisation and RBP binding; impact on synaptic and immune genes	APP/PS1 models (AD-like pathology with developmental relevance)	Mouse cortex; methylated circRNA profiling	[57]
Rett-associated circRNAs/T-UCRs	Chromatin and synaptic-network regulation within the MeCP2-controlled landscape	Rett syndrome models (mouse and human)	Mouse models; human datasets; integrative ncRNA profiling	[58]
hsa_circ_CORO1C–miR-708-3p–JARID2/LNPEP	Epigenetic regulation via JARID2; neurodevelopmental signalling	First-episode schizophrenia	Human brain tissue; region-specific circRNA modules	[59]
Schizophrenia circRNA modules	Convergence on dopaminergic and neuroinflammatory pathways	First-episode schizophrenia	Whole-transcriptome analysis in independent cohorts	[60]
EV circRNA ceRNA networks	Neuronal adhesion and oxidative-stress regulation via circRNA–miRNA–mRNA modules	Early-onset schizophrenia/neuropsychiatric risk	Human plasma extracellular vesicles: clinical samples	[61]
ERVWE1–circ_0001810–AK2 pathway	Retroviral envelope activation, circ_0001810 upregulation, mitochondrial dysfunction via AK2	Schizophrenia/neurodevelopmental vulnerability	Human cell models/clinical samples	[62]
circRtn4–miR-24-3p–CHD5 axis	Derepression of CHD5; neurite outgrowth and chromatin remodelling	Normal brain development	Mouse neurons/conserved human loci	[63]
Edis–Relish–castor feedback loop	Integration of innate immunity with neuronal differentiation	Drosophila neurodevelopment and immune response	Invertebrate models; genetic and molecular studies	[64,65]
circFGFR2	Astrocytic pyroptosis via inflammasome activation	Ischaemic stroke/neuroinflammatory brain injury	Mouse models/astrocyte assays	[66]
hsa_circ_0000288–Caprin1 axis	Stabilisation of Caprin1; preservation of synaptic integrity	Epilepsy	Human tissue/cell studies	[67]
circ_Csnk1g3	Promotion of hippocampal necroptosis and inflammation	Epilepsy/hippocampal injury	Mouse epilepsy models	[67]
circ_0049472–miR-22-3p–PDE4A	Regulation of PDE4A; oxidative stress and RNA-decay pathways	AD–NDD spectrum	Human and/animal models	[68]

ASD, autism spectrum disorder; AD, Alzheimer’s disease; NDD, neurodevelopmental disorder; CP, cerebral palsy; DE, differentially expressed; circQTL, circular RNA quantitative trait locus; PM2.5, particulate matter ≤ 2.5 μm; EV, extracellular vesicle; MAPK, mitogen-activated protein kinase; CHD5, chromodomain helicase DNA-binding protein 5; BCL11A, B-cell lymphoma/leukaemia 11A; MEF2C, myocyte enhancer factor-2C; T-UCR, transcribed ultraconserved region; APP/PS1, amyloid precursor protein/presenilin-1 transgenic mouse model; m^6^A, N^6^-methyladenosine; JARID2, Jumonji and AT-rich interaction domain-containing 2; LNPEP, leucyl/cystinyl aminopeptidase; AK2, adenylate kinase 2; PDE4A, phosphodiesterase 4A.

**Table 4:** Summary of key tsRNA and piRNA species, their molecular mechanisms, and disease relevance.

Small RNA	Biological Mechanism	Disease	Study Type	References
5′-tiRNAs	Inhibit cap-dependent translation via eIF4F displacement; metabolic stress adaptation.	Neurodevelopment under oxidative stress	Cell models/mouse brain	[69]
tRNA hypomethylation (NSUN2/DNMT2 loss)	m^5^C-tRNA destabilization-tsRNA generation-impaired neuronal differentiation	Microcephaly, ID, NDD phenotypes	Mouse models/human mutation cases	[70]
m^1^A/m^7^G-dependent tsRNA sorting into AGO	Codon-biased translational control; modulation of MAPK/mTOR signalling	Neurodevelopment/plasticity	Biochemical assays, neuronal cultures	[70,71]
Aged-sperm tsRNA repertoire	Intergenerational transmission alters offspring neurogenesis & behaviour	NDD-relevant behavioural traits	Mouse sperm injections; controlled experiments	[72]
Synthetic/therapeutic tsRNAs	Target-specific translational repression or rescue	Translational NDD models	Preclinical in vitro/in vivo	[73]
piRNA–PIWIL1/PIWIL2 pathway	Regulates dendritic spine morphology, LTP, and synaptic strength	Cognition and memory	Mouse hippocampus; knockdown/KO models	[74]
Brain-enriched piRNAs in ASD	Synaptic, immune, and mitochondrial pathway regulation	Autism spectrum disorder	Human plasma/stool small-RNA seq	[75,76,77]
Nuclear piRNA–DNMT/HMT axis	Chromatin modification, long-term synaptic-gene silencing	General NDD relevance	Cell/animal models	[74]
Cytoplasmic piRNAs binding long 3′-UTRs	Local translation control; structural plasticity	Synaptic maturation/circuits	Neuronal cultures	[74]
Gut-derived bacterial tRNA fragments	Immune pathway modulation-neural development effects	ASD/NDD risk	Human stool/serum analyses	[76]
NSUN2/TRMT6/61/METTL1 mutations	Disruption of tRNA methylation-widespread tsRNA/piRNA imbalance	Microcephaly, ID, ASD-like traits	Human cases/mouse models	[74,70]
HEN1/Zucchini pathway defects	Aberrant piRNA maturation-transposon derepression-neural dysfunction	NDD-like phenotypes	Animal models	[74]

NDD, neurodevelopmental disorder; ID, intellectual disability; ASD, autism spectrum disorder; KO, knockout; LTP, long-term potentiation; tsRNA, tRNA-derived small RNA; tiRNA, tRNA-derived stress-induced RNA; m^5^C, 5-methylcytosine; m^1^A, N^1^-methyladenosine; m^7^G, 7-methylguanosine; AGO, Argonaute protein; piRNA, PIWI-interacting RNA; PIWIL1/2, PIWI-like protein 1/2; DNMT, DNA methyltransferase; HMT, histone methyltransferase; NSUN2, NOP2/Sun RNA methyltransferase 2; TRMT6/61, tRNA methyltransferase 6/61 complex; METTL1, methyltransferase-like protein 1; HEN1, HUA ENHANCER 1 methyltransferase; 3′-UTR, 3′ untranslated region; PM2.5, particulate matter ≤ 2.5 μm.

**Table 5:** Circulating ncRNAs as biomarkers in NDDs and related disorders.

Disease	Biofluid	ncRNA Class	Direction of Change	References
ASD	Serum	miR-195-5p	Up-regulated	[7]
ASD	Serum	miR-499a-5p	Up-regulated	[8]
ASD	Plasma	Age-associated miRNA panel (miR-4433b-5p, miR-15a-5p, miR-335-5p, miR-1180-3p)	Mixed age-dependent shifts	[23]
ASD	Peripheral blood	lncRNAs PCAT-29, LINC-PINT, lincRNA-p21, lincRNA-ROR, PCAT-1	Down-regulated	[35]
ASD	Plasma EVs	miR-215-5p	Down-regulated	[6]
ASD	Plasma EVs	circRNA panel (circRNA–miRNA–mRNA network)	Multiple circRNAs are differentially expressed	[53]
ASD	Stool and serum	piRNAs and miRNAs (gut–brain axis)	Co-altered with microbiota composition	[75,76,77]
ADHD	Whole blood/plasma	Panels of ~29 miRNAs (miR-26b-5p, miR-185-5p, miR-142-3p)	Mixed	[13,14,15,25]
Cerebral palsy	Peripheral blood	hsa_circ_0086354	Differentially expressed	[55]
Schizophrenia	Plasma EVs	circRNA-mediated ceRNA networks	Multiple circRNAs altered	[61]

ASD, autism spectrum disorder; ADHD, attention-deficit/hyperactivity disorder; EV, extracellular vesicle; miRNA, microRNA; lncRNA, long non-coding RNA; circRNA, circular RNA; piRNA, PIWI-interacting RNA.

**Table 6:** Therapeutic strategies targeting ncRNA pathways.

Therapeutic	ncRNA	Disease	Delivery	References
miRNA mimic/replacement	miR-137	ASD-like phenotypes in rodent models	RVG-engineered extracellular vesicles	[22]
miRNA mimic/replacement	miR-215-5p	VPA-induced ASD mouse model	Viral vectors or EVs (preclinical)	[6]
Antagomirs/ASO inhibitors	miR-34a-5p	Epilepsy and hippocampal ferroptosis models	Chemically modified ASOs	[17]
ASO-mediated knockdown	NEAT1	Rett syndrome; VPA-induced ASD models	Gapmer ASOs; viral vectors	[34,50]
ASO knockdown/CRISPR interference	UBE3A-ATS	Angelman syndrome mouse models	AAV–dCas9 constructs; ASOs	[46]
siRNA/shRNA-mediated knockdown	circ_0001810, circ-Csnk1g3	Schizophrenia; epilepsy models	Viral vectors; nanoparticle delivery	[62]
Overexpression of protective circRNA	hsa_circ_0000288	Epilepsy mouse models	Viral overexpression vectors	[67]
Modulation of tRNA-modifying enzymes	NSUN2, TRMT6/61, METTL1/WDR4	Microcephaly, ID, neurodevelopmental deficits	Small molecules or gene therapy	[70,74,79]
Synthetic tsRNA mimics	tRFAla-AGC-3-M8	Alzheimer’s disease and neuroinflammation models	Chemically stabilised RNA mimics	[79]
PIWI–piRNA pathway enhancement	Protective neuronal piRNA clusters	Parkinson’s disease; neurodegenerative models	Gene therapy; small-molecule PIWI modulators	[74,80,81]
CRISPR–dCas9 epigenetic editing	Enhancers, promoters, and lncRNA loci	NDD risk loci in ASD and syndromic disorders	AAV-delivered dCas9–KRAB/VP64 systems	[46]

ASD, autism spectrum disorder; VPA, valproic acid; ASO, antisense oligonucleotide; RVG, rabies virus glycoprotein peptide; UBE3A-ATS, UBE3A antisense transcript; AAV, adeno-associated virus; dCas9, nuclease-dead CRISPR-associated protein 9; KRAB, Krüppel-associated box repressor domain; VP64, viral protein 64 transcriptional activator; siRNA, small interfering RNA; shRNA, short hairpin RNA; circRNA, circular RNA; tsRNA, tRNA-derived small RNA; piRNA, PIWI-interacting RNA; PIWI, P-element induced wimpy testis protein family; ID, intellectual disability; NSUN2, NOP2/Sun RNA methyltransferase 2; TRMT6/61, tRNA methyltransferase 6/61 complex; METTL1/WDR4, methyltransferase-like protein 1/WD-repeat domain 4.

## Data Availability

No new data were created or analysed in this study.
